# Influence of Malnutrition on Outcome after Hip Fractures in Older Patients

**DOI:** 10.3390/jpm13010109

**Published:** 2023-01-03

**Authors:** Michael Millrose, Wolfgang Schmidt, Julian Krickl, Till Ittermann, Johannes Ruether, Hermann-Josef Bail, Markus Gesslein

**Affiliations:** 1 Department of Trauma Surgery and Sports Medicine, Garmisch-Partenkirchen Medical Centre, 82467 Garmisch-Partenkirchen, Germany; 2 Department of Orthopedics and Traumatology, Paracelsus Medical University, 90471 Nuremberg, Germany; 3 Institute for Community Medicine, SHIP/Clinical-Epidemiological Research, University of Greifswald, 17489 Greifswald, Germany

**Keywords:** malnutrition, hip fractures, geriatric trauma, sarcopenia

## Abstract

Background: Malnutrition might lead to a worse outcome in hip fractures of older patients. The purpose of this study is to analyze different indicators of malnutrition that lead to worse outcomes. Methods: 252 patients of a geriatric trauma unit were analyzed prospectively. Different demographic, as well as data on the trauma and whether osteoporosis prophylaxis or anticoagulation was present, were recorded. The nutritional status with respect to laboratory parameters as well as nutritional risk score was also analyzed. Results: The main finding of this study is that a poor nutritional status is statistically significantly associated with higher mortality as well as worse independence (*p* < 0.05). A postoperatively prescribed osteoporosis prophylaxis is protective of mortality and independence (*p* < 0.05). Conclusions: Malnutrition of geriatric patients increases the risk for death, worse mobility, and independence after hip fractures. Osteoporosis prophylaxis prescribed during an inpatient stay enables patients to retain their independence. The nutritional status of geriatric patients with hip fractures should be obtained and provisions made.

## 1. Introduction

Among older hospitalized people, malnutrition or risk of malnutrition shows a high prevalence—as high as 40% [1]. Malnutrition is defined by the World Health Organization as “deficiencies, excesses or imbalances in a person’s intake of energy and/or nutrients” [2]. In different studies it could be shown that malnutrition increases hospitalization, length of hospital stays as well as the overall mortality [3,4]. It also contributes to muscle wasting and, therefore, reduced muscular power and mobility [5].

One of the most important acute health risk factors in older ages are hip fractures (HF); their incidence increases significantly with age in the German population over 65 years of age by nearly six-fold with an additional increased risk of dying of almost nineteen-fold [6,7]. It is well known that HFs in geriatric patients often cause severe functional impairment with increased disability, reduction of quality of life, and by that, consequently loss of autonomy. They also increase the mortality of this special group [8].

In a systematic review of Foo and colleagues they could provide evidence that malnutrition in patients with a hip fracture is significantly associated with increased mortality after 12 months as well as functional dependence over a longer time period with a trend to more supported living arrangements, nursing homes, etc. [1]. Therefore, HFs in malnourished older people pose an especially great threat to the individual patient as well as the health care system itself due to high costs and the risk of overburdening the nursing homes.

The overall aim would be that by addressing the malnutrition of an old patient with a HF, the clinical outcome might be improved. The malnutrition can be assessed by clinical as well as laboratory parameters. Even subclinical deficits of nutrients can have an impact on the outcome if supplemented. Additionally, the connection between malnutrition and the development of osteoporosis is well described [9]. Due to the demographic changes, the population gets older and, therefore, the prevalence of HF in this special group will continuously grow [10].

In this study we wanted to present the results of mortality, functional outcome, and independence of our geriatric trauma unit and analyze different risk factors in terms of the trauma itself and especially different indicators for malnutrition leading to a worse outcome.

## 2. Materials and Methods

This prospective study was conducted from January 2018 to September 2020 at a regional care hospital with a department for geriatric trauma surgery. During the entire study period, 916 patients were admitted with a trauma diagnosis. All patients suffering from a proximal femoral fracture, regardless of traumatic or pathological genesis, were asked to participate in this study. The traumatic etiology consisted mostly of falls, either because of an external reason such as slipping/tripping or internal reasons such as syncope. Out of the 916 admitted patients, 269 patients could be included in the study. Seventeen study participants were lost during the follow-up and 252 patients (69 male and 183 female) could be assessed in the analysis.

The inclusion criteria of the study were a minimum age of 65 years and a performed osteosynthesis of the femur or joint replacement of the hip, regardless of the implant used. Exclusion criteria of this study were lack of consent or ability to consent to the study. Data sets which lack 3 or more items were also discarded.

This study was conducted according to the guidelines of the declaration of Helsinki and the analysis and publication of the data was approved by the Institutional Review Board of Paracelsus Medical University (protocol code IRB-2022-018; approved on 23 November 2022). Written informed consent was obtained from every participant or their respective legal custodian.

At inclusion, the demographic parameters age, sex, American Society of Anesthesiologists (ASA) classification and living situation, the type of fracture, preoperative osteoporosis therapy as well as anticoagulation and preoperative walking ability were recorded. The time between admission and operation as well as the operation procedure were obtained. In addition to the surgery procedure itself, closed versus open procedures were differentiated. The outcome parameters were mortality during the acute inpatient stay as well as after 120 days and a change in the patient’s location; the ability to walk was recorded during follow-up 120 days after admission.

For assessing the nutritional status of the included patients, the body mass index (BMI), serum albumin level, adjusted calcium as well as levels of vitamin B12, vitamin D and folic acid in serum were analyzed. The lower threshold value for those blood parameters were albumin 3.2 g/dL, calcium 4.4 mval/L, vitamin B12 197 pg/mL, vitamin D 20 ng/mL and folic acid 4.60 ng/mL, which were the lower margins for the standard range of the respective parameters provided by our laboratory. The nutritional risk score during the inpatient stay was recorded by nutritionists [11].

The data were obtained prospectively and analyzed retrospectively. The statistical analysis was performed using STATA 17.0 (Stata Corporation, College Station, TX, USA). Characteristics of the study population are reported and stratified by sex as the mean and standard deviation for continuous variables and as absolute numbers and percentages for categorical variables. Associations of potential risk factors with inner-clinic death, death after 120 days, worsened mobility after surgery and worsened domicile after surgery were analyzed by logistic regression models adjusted for age and sex. Results are reported as odds ratio, 95% confidence interval and *p*-value. A *p* < 0.05 was considered as statistically significant.

## 3. Results

During this prospective study 252 patients could be analyzed. They suffered from a hip (proximal femur) fracture and were treated at the geriatric trauma surgery department. The study population consisted of 183 females with a mean age of 84.38 ± 7.25 years and 69 men with a mean age of 83.78 ± 5.91 years. The mean BMI of all patients was 23.79 ± 3.86 with 19 underweight, 144 healthy weight, 69 overweight and 20 obese (Table 1). Of the analyzed 252 patients, 3 were categorized ASA 1, 46 ASA 2, 181 ASA and 22 ASA 4.

During inpatient care 22 patients died; during the post-discharge follow-up period of 120 days after surgery the number doubled to 44 patients. The details of the two outcome parameters (mobility and independence) are depicted in Table 2.

With respect to the mobility of the 208 patients analyzed at follow-up, 18 were able to improve their mobility, 85 stayed at the same mobility level as before the fracture, while 105 patients achieved a worse mobility level, meaning that they needed additional walking support.

Concerning the independence of the living situation, 6 patients were living more self-reliant, 180 lived as before and 26 were newly nursing home depended.

The risk factors which were included into the analysis are shown in Table 3 in detail. The time between admission and surgery was recorded and showed that 90 patients were operated on within 6 h, 44 within 12 h and 83 within 24 h. Only 35 had a time span of over 24 h.

The NRS was obtained by nutritionists in 158 patients with a median of 3. The serum albumin level showed a mean of 2.76 ± 0.45 g/dL (n = 227) with 211 patients below the normal range of 3.5–5.2 g/dL. The total calcium was 4.52 ± 0.25 mval/L (n = 252) with only 9 patients below the threshold of 4.4 mval/L. The mean vitamin B12 level was 469.40 ± 418.25 pg/mL (n = 235) with 47 patients below and 37 patients above the normal range of 191–771 pg/mL; the mean vitamin D level was 20.81 ± 20.14 ng/mL (n = 236) with 136 patient showing levels below the threshold of 20 ng/mL. Folic acid showed a mean of 7.68 ± 6.19 ng/mL (n = 230) with 89 patients below 4.6 ng/mL (Table S1).

During the inpatient stay at the geriatric trauma surgery department, 43 patients received oral nutritional supplements.

The results of the regression analysis are shown in Table 4. They imply that the use of anticoagulants before the trauma is statistically significantly correlated with a higher inner clinic mortality (*p* < 0.05). The prescription of osteoporosis prophylaxis at discharge is associated with a lower mortality as well as better or similar independence at follow-up (*p* < 0.05 respectively). A high perioperative risk (ASA 4) elevates the risk of death at follow-up significantly (*p* < 0.05). A poor nutritional status of the geriatric patients in terms of NRS is associated with a higher mortality (*p* < 0.05) and worse independence (*p* < 0.05). All blood parameters show a trend that levels below the lower threshold value increase the mortality and worsen the mobility and independence.

## 4. Discussion

The most important finding of this study is the influence of malnutrition on outcomes after a HF. Results showed that a higher NRS (i.e., poor nutritional status) is associated with a higher mortality during the in-patient stay as well as at the follow-up 120 days after the trauma. Additionally, the independence (significant) and the mobility (on trend) of the older patient are worse with malnutrition. The prescription of osteoporosis prophylaxis shows a lower mortality and a better level of independence.

HFs in fragile and/or elderly patients causes a serious risk for their health and their independence. In the present study, it could be shown that nearly two thirds of the included patients worsened in their mobility—mostly to rollator mobility. Additionally, the number of patients that were bedridden more than doubled. The number of patients in nursing homes increased by 26, which poses a great deterioration in their independence. The independence, with respect to activities of daily life, degrades also, since there are different tasks that one is not able to perform with a rollator. Our results are in concordance with the systematic literature review by Peeters et al. [8]. They showed that HFs had a negative impact on the overall health status as well as health-related quality of life of elderly patients (>65 years old), especially the health status in terms of physical, psychological and social aspects was seriously affected. They showed that the literature has evidence that the pre-fracture condition with respect to physical functioning or nutritional status seems to have a negative impact on the outcome. They concluded that optimizing nutritional intake and physical rehabilitation should be recommended after surgery; however, there were only few studies that examined its effectiveness [8].

A recent systematic review by Foo, Wong and Lew from 2021 analyzed the prevalence of malnutrition and its associated outcomes after a HF in a geriatric population [1]. They showed that the prevalence of malnutrition in those patients ranged from 4.0–39.4%. In our study we found that in 158 patients, most of them were at least at risk for malnutrition. They showed that malnourished older patients were significantly at risk for functional dependence, which is in concordance with our results since nearly 50% achieved a worse mobility level at the 120-day follow-up. From their results, they raised the question whether nutritional support would improve the clinical outcome. Our results of the association of malnutrition with higher mortality, as well as worse independence are in concordance with their results. We were not able to prove that a dietary supplementation improves the outcome. This could be due to the lack of consequences in prescribing these, which in our geriatrics department is a medical duty.

The systematic review by Malafarina et al. from 2018 showed that mortality was inversely associated with albumin levels [12]. They found in the literature a relative risk of dying of 1.52 with hypalbuminemia. These findings are shown in trend by our results with an odds ratio of 1.93 of death at follow-up with lower albumin levels. This systematic review also confirmed other findings of our study, such as the association of malnutrition with higher mortality and worse independence.

A clinical study form 2015 in Germany showed that malnutrition has a negative impact on the regaining of their previous Barthel index for the activities of daily living or their preadmission mobility level [13]. The nutritional status of the included patients was analyzed by the Mini Nutritional Assessment (MNA). The MNA in contrast to the NRS used in this study additionally includes measurements of anthropometric data, which might show a clearer picture of the overall status of the geriatric patient since it includes data on the muscle mass and strength respective to their decline (sarcopenia). We have used the NRS in combination with sarcopenia screening, which mostly confirmed the malnourishment with probable sarcopenia (see Table S1). A total of 45% of patients of the study by Goisser et al. were at risk of malnutrition or were malnourished. This shows the same tendency as our study, whereby nearly half of all geriatric patients are not well-nourished. They concluded that a worse preadmission nutritional status is associated with a worse functional outcome. This agrees with our results.

It is well published in the literature that malnutrition of the elderly patient is playing an important part in developing osteoporosis [14,15]. Besides pharmacological agents, nutritional support as well as fall prevention strategies and exercise can be utilized to treat osteoporosis and prevent further osteoporotic fractures, such as hip or vertebral fractures. The sufficient treatment of malnutrition and the normalization of the related blood parameter can improve the outcome of elderly patients with osteoporosis and should be routinely implemented during an in-patient stay after an osteoporosis-defining fracture. In our study we could show that more than half of the study population suffered from a deficient vitamin D level. At discharge, nearly all of the treated patients were supplemented with vitamin D and calcium to compensate this deficiency after an osteoporotic fracture.

One of the strengths of our study is that the nutritional status of our patients was routinely supported by specific parameters, for example, albumin. Cabrerizo et al. stated that albumin is a good marker of nutritional status [16]. Additionally, the NRS was obtained in our study by nutritionists. In a clinical study by Benoit et al. from 2015, it could be shown that the assessment of the nutritional status by surgeons was significantly worse than by nutritionist. Therefore, the data obtained in our study more likely represents the actual status of the included patient.

A limitation of our study is the follow-up period of only 120 days. Peeters et al. showed in their systematic review that the health status of the geriatric patient after a HF improved during the first 6 months; therefore, the reported outcomes in terms of mobility and to a lesser degree independence might be worse after 6 months or even longer of rehabilitation. Another limitation of our study is the only sparsely provided dietary supplement. A randomized controlled study with or without dietary supplement to examine the outcome after proximal femur fracture in the elderly patient is currently planned. Additionally, confounding diagnoses, such as chronic obstructive pulmonary disease or diabetes, were not recorded and might have an influence on the nutritional status and outcome after a HF.

Further high quality randomized controlled studies are needed to provide evidence that dietary supplementation improves the malnutrition of geriatric patients and, therefore, lowers the risk of mortality.

## 5. Conclusions

Geriatric patients with malnutrition, shown by the nutritional risk score as well as blood test parameters, have a higher risk of dying during the first 120 days after surgical treatment of a HF. Even though this study could not provide evidence that dietary supplements would improve the outcome, taking appropriate steps to improve the nutritional status of the older patient should be considered. The prescription of osteoporosis prophylaxis retains independence and should be routinely suggested.

## Figures and Tables

**Table 1:** Demographics of study population.

	Female [n = 183]	Male [n = 69]
Age [years]	84.38 (±7.25)	83.78 (±5.91)
Height [m]	1.63 (±0.07)	1.76 (±0.07)
Weight [kg]	62.79 (±11.45)	75.51 (±11.07)
BMI	23.56 (±4.02)	24.39 (±3.33)

All data is presented as mean with standard deviation.

**Table 2:** Details of the two outcome parameters: mobility and independence.

Mobility	Independent	Cane/Crutch	Rollator	Only Indoor	Bedridden
Preadmission [n = 252]	91	27	58	68	8
Follow-up 120d [n = 208]	33	28	72	53	22
**Independence**	Own home	Nursing home	hospital		
Preadmission [n = 252]	198	54			
Follow-up 120d [n = 208]	158	48	2		

**Table 3:** Details of risk factors included in the statistical analysis.

**Anticogulation**	None	OAC	PAI	NOAC	combination
[n = 252]	122	18	57	44	11
**Osteoporosis prophylaxis**	None	Vit. D + calcium	Specific	combination	
Preadmission [n = 252]	195	52	1	4	
Discharge [n = 230]	12	172	1	47	
**Implant** [n = 252]	DHS	Nail (CRIF)	Nail (ORIF)	THR	ORIF
	13	121	23	83	12

OAC: oral anticoagulants; PAI: platelet aggregation inhibitors; NOAC: novel oral anticoagulants; specific osteoporosis prophylaxis: bisphosphonates, estrogen agonist/antagonist, etc.; DHS: dynamic hip screw; nail (CRIF): closed reduction and internal fixation with trochanteric nail; nail (ORIF): open reduction and internal fixation with trochanteric nail; THR: total hip replacement; ORIF: open reduction and internal fixation

**Table 4:** Results of regression analysis of risk factors.

	Death Inner Clinic OR (95%-CI)	Death after 120 Days OR (95%-CI)	Worsened Mobility OR (95%-CI)	Worsened Independence OR (95%-CI)
Anticoagulant use (preadmission)	3.29 (1.12; 9.69) *	1.78 (0.87; 3.65)	1.05 (0.63; 1.77)	1.10 (0.60; 2.00)
Osteoporosis medication (preadmission)	1.57 (0.58; 4.23)	1.29 (0.60; 2.79)	0.90 (0.48; 1.67)	1.02 (0.51; 2.03)
Osteoporosis medication (at discharge)	0.10 (0.04; 0.29) *	0.19 (0.08; 0.43) *	0.75(0.33; 1.69)	0.37 (0.16; 0.82) *
ASA risk classification				
3 vs. (1,2)	2.74 (0.33; 23.0)	7.13 (0.91;55.6)	0.89 (0.44; 1.77)	1.73 (0.65; 4.57)
4 vs. (1,2)	9.27 (0.95; 91.0)	18.2 (2.01; 164) *	0.93 (0.31; 2.78)	3.25 (0.92; 11.4)
Body mass index	0.90 (0.78; 1.04)	0.89 (0.80; 0.99) *	0.99 (0.93; 1.06)	0.94 (0.87; 1.03)
Nutritional risk score	2.17 (1.37; 3.43) *	1.68 (1.16; 2.41) *	1.10 (0.80; 1.53)	1.59 (1.13; 2.22) *
Low albumin	3.19 (0.39; 26.0)	1.93 (0.53; 7.10)	2.21 (0.70; 7.00)	1.55 (0.57; 4.17)
Low calcium	1.55 (0.57; 4.17)	2.33 (1,12; 4.85) *	1.67 (0.92; 3.04)	1.53 (0.80; 2.91)
Low vitamin B12	1.32 (0.39; 4.50)	0.88 (0.34; 2.23)	1.18 (0.61; 2.30)	1.46 (0.70; 3.05)
Low vitamin D	1.41 (0.46; 4.29)	0.92 (0.43; 1.97)	1.26 (0.73; 2.17)	0.84 (0.44; 1.58)
Low folic acid	1.36 (0.47; 3.96)	2.10 (0.97; 4.51)	1.15 (0.65; 2.04)	1.83 (0.96; 3.46)
Dietary supplement	0.23 (0.03; 1.79)	1.38 (0.58; 3.29)	1.11 (0.56; 2.21)	1.21 (0.57; 2.57)

Logistic regression models adjusted for age, sex and degree of care. Lower threshold value: albumin 3.2 g/dL; calcium 4.4 mval/L; Vit. B12 197 pg/mL; vitamin D. 20 ng/mL; and folic acid 4.60 ng/mL. OR: odds ratio; CI: confidence interval; statistical significance level * *p* < 0.05.

## Data Availability

The data of our study population is provided in the supplementary material Table S1.

## Supplementary Materials

The following supporting information can be downloaded at https://www.mdpi.com/article/10.3390/jpm13010109/s1: Table S1: Data of included patients.
