# Supplementary material for: Influence of Malnutrition on Outcome after Hip Fractures in Older Patients
Source: J Pers Med. 2023 Jan 3;13(1):109. doi: 10.3390/jpm13010109 (PMC9862302; doi:10.3390/jpm13010109)
Supplement: Supplementary file 1 [file jpm-13-00109-s001.zip › jpm-2147244-supplementary.pdf]

Nr.	DoB	Age	Sex [1=female; 2=male]	Admission [date]	Domicile [1=own home; 2=nursing home]	Anticoagulant [0=none;1=OAC;2=ASS/Plavix; 3=NOAC; 4=combination]	Osteoporosis medication [0=none;1=Vit. D + calcium;2=specific;3=combination]	Mobility prefracture [0=independent; 1=cane/crutch; 2=rollator; 3=only indoor; 4=bedridden]	ASA [1-4]	Surgery [date]	Anesthesia [1=general; 2=other]	Implant [1=DHS,2=nail,3=nail open,4=THR,5=osteosynthesis]	Mobility 7th pod [0=independent; 1=cane/crutch; 2=rollator; 3=walker; 4=bedridden]	Osteoporosis medication [0=none; 1=Vit D + calcium; 2=specific; 3=combination]	Discharge [1=home;2=nursing home;3=geriatric rehabilitation; 4=rehabilitation; 5=hospital;6=death]	discharge postoperatively [d]	Readmission [0=no;1=yes]	Domicile [1=home;2=nursing home;3=hospital; 4=death]	Difference Domicile [1=better;2=same;3=worse]	Mobility 120d postOP [0=independent; 1=cane/crutch; 2=rollator; 3=only indoor; 4=bedridden;5=not applicable]	Difference Mobility [1=better;2=same;3=worse]	Height [m]	Weight [kg]	BMI	Nutritional risk score	Albumin [g/dl]	Total Protein [g/dl]	Cholinesterase [U/l]	Total calcium [mval/l]	Vit. B12 [pg/ml]	Vit. D [ng/ml]	Folic acid [ng/ml]	Dietary supplement
1	26.07.40	77	1	03.01.18	1	0	0	1	2	03.01.18	1	3	2	3	1	19	0	1	2	1	2	1,5	49	21,78	-	3,19	6,76	7964	4,58	121,7	47	4,91	Fortimel compact
2	30.04.36	81	1	03.01.18	1	0	0	0	2	03.01.18	1	2	2	1	3	16	0	1	2	1	3	1,78	57	17,99	-	2,94	7,04	6022	4,64	217,7	4	8,01	-
3	04.08.36	81	2	03.01.18	1	0	0	0	2	04.01.18	1	2	2	1	1	16	0	1	2	1	3	1,7	72	24,91	-	2,89	7,34	6284	4,42	342,7	3	2,76	-
4	24.09.30	87	2	04.01.18	1	2	0	2	3	04.01.18	1	2	2	1	3	18	0	1	2	2	2	1,66	61	22,14	-	3,37	5,9	6099	4,5	195,9	25	3,11	-
5	08.01.46	72	1	12.01.18	1	0	0	1	2	13.01.18	1	2	2	0	1	15	0	1	2	1	2	1,72	84	28,39	-	2,91	7,36	8786	4,54	767	21	12,28	-
6	09.12.43	74	1	16.01.18	1	0	0	0	1	16.01.18	1	2	2	1	3	22	0	1	2	0	2	1,55	50	20,81	-	2,77	7,27	3975	4,42	528,7	3	5,14	Fresubin
7	06.03.36	81	1	17.01.18	1	0	0	0	3	17.01.18	1	2	2	1	3	23	0	1	2	2	3	1,52	50	21,64	-	3	7,41	6769	4,32	914,7	6	4,7	ProvideXtra
8	27.07.38	79	1	23.01.18	1	0	3	0	2	25.01.18	1	4	2	3	1	14	0	1	2	1	3	1,6	50	19,53	-	2,45	6,29	6577	4,08	495,6	3	7,21	Fortimel compact
9	21.10.21	96	1	25.01.18	2	1	0	3	4	25.01.18	1	1	4	0	6	5	0	4	3	5	3	1,55	50	20,81	-	2,04	6,35	4416	4,22	443,2	3	2,4	-
10	25.12.34	83	1	15.01.18	1	2	0	1	3	26.01.18	1	4	2	1	3	20	0	1	2	1	2	1,64	80	29,74	-	2,97	6,24	5562	4,18	791,1	3	4,6	-
11	02.03.35	82	1	29.01.18	1	2	0	2	3	29.01.18	1	2	2	1	2	16	0	1	2	2	2	1,62	52	19,81	-	2,62	6,61	5695	4,5	321,4	4	4,08	Fortimel compact
12	12.06.28	89	1	29.01.18	1	0	1	0	3	29.01.18	1	2	2	1	3	15	0	1	2	1	3	1,58	86	34,45	-	2,18	6,64	5504	4,22	191,2	8	3,64	Fortimel compact
13	14.06.29	88	1	05.02.18	1	0	0	2	4	05.02.18	1	4	2	1	6	75	0	4	3	5	3	1,67	78	27,97	-	3,1	6,39	4688	4,16	301,9	3	3,01	Fortimel compact
14	26.08.30	87	2	08.02.18	1	0	0	3	3	08.02.18	1	2	3	0	1	11	0	4	3	5	3	1,65	65	23,88	-	2,63	5,6	4708	4,32	317,3	22	3,21	-
15	14.03.41	76	2	13.02.18	1	2	1	2	4	13.02.18	1	2	4	0	6	5	0	4	3	5	3	1,75	70	22,86	-	2,98	7,62	5463	4,64	322,5	3	4,68	-
16	29.08.22	95	1	15.02.18	2	2	1	2	3	15.02.18	1	3	2	1	2	16	0	2	2	2	2	1,6	50	19,53	-	3,17	7,04	8399	4,68	466	33	31,18	-
17	06.11.32	85	2	19.02.18	1	3	0	1	3	19.02.18	1	2	2	1	1	15	0	1	2	1	2	1,68	63	22,32	-	3,15	6,74	4255	4,58	300,4	6	3,6	-
18	07.08.34	83	1	28.02.18	1	0	0	0	3	01.03.18	1	4	2	1	1	16	0	1	2	2	3	1,54	60	25,30	-	2,94	8,37	4970	4,5	764,4	19	21,2	-
19	08.04.31	87	1	10.03.18	2	0	0	3	3	10.03.18	1	4	2	1	2	19	0	2	2	2	1	1,76	60	19,37	-	2,42	6,68	6888	4,8	307,9	24	4,08	-
20	01.01.40	78	1	13.03.18	1	0	1	0	2	14.03.18	1	5	1	1	1	17	0	1	2	2	3	1,63	72	27,10	-	3,26	7,1	6647	4,68	601,2	32	8,54	-
21	17.01.20	98	1	23.03.18	1	0	0	1	3	23.03.18	1	2	2	1	3	20	0	1	2	2	3	1,65	75	27,55	-	4,01	7,38	6201	4,9	-	-	-	-
22	13.01.17	101	1	25.03.18	2	3	0	3	4	29.03.18	1	2	2	0	2	15	0	2	2	3	2	1,6	45	17,58	-	2,75	7,09	6773	4,52	178,2	6	3,54	Energea P + Fortimel Comp
23	25.07.40	77	1	04.04.18	1	0	0	2	3	04.04.18	1	2	2	1	3	22	1	1	2	4	3	1,62	70	26,67	-	-	6,68	6748	4,32	267,1	3	2,88	-
24	02.12.33	84	1	06.04.18	1	0	0	0	2	06.04.18	1	2	2	1	3	24	0	1	2	2	3	1,6	50	19,53	-	-	7,05	6663	4,8	1627	12	9,31	-
25	23.06.34	84	1	10.04.18	1	0	0	0	3	11.04.18	1	4	0	3	3	17	0	1	2	0	2	1,58	60	24,03	-	2,58	6,91	8277	4,86	264,2	7	3,55	-
26	28.07.28	89	1	12.04.19	1	0	1	1	4	12.04.18	1	3	2	1	3	18	1	1	2	2	3	1,6	60	23,44	-	2,53	6,61	7297	4,56	232,5	35	6,7	-
27	11.05.28	89	1	14.04.18	2	0	0	3	3	14.04.18	1	2	3	0	2	12	0	2	2	3	2	1,43	48	23,47	-	-	7	6535	4,46	107,9	3	4,65	-
28	13.08.30	87	1	15.04.18	2	2	0	2	3	16.04.18	1	4	2	1	2	17	0	2	2	3	3	1,61	54	20,83	-	2,46	7,23	7735	4,72	245,1	4	3,97	Fortimel compact
29	07.08.32	85	1	18.04.18	1	4	0	3	3	19.04.18	1	4	2	1	2	19	0	2	3	4	3	1,72	76	25,69	-	1,83	7,36	5948	4,08	124,7	3	2,94	Fresubin
30	02.01.30	88	1	20.04.18	2	0	0	0	2	21.04.18	1	1	1	3	1	18	0	1	1	0	2	1,62	75	28,58	-	3,1	6,91	7643	4,62	564,7	10	-	-
31	20.04.33	85	2	23.04.18	2	2	1	2	3	24.04.18	1	3	4	0	6	12	0	4	3	5	3	1,8	82	25,31	-	2,72	6,47	5886	4,58	240,8	38	1,51	-
32	06.08.29	88	1	23.04.18	2	1	0	4	3	24.04.18	1	5	4	0	2	23	0	2	2	4	2	1,72	64	21,63	-	2,58	7,93	8458	5,2	456,7	35	16,97	-
33	03.09.33	84	1	23.04.18	2	0	0	3	3	25.04.18	1	4	4	1	2	11	0	2	2	4	3	1,6	55	21,48	-	2,38	6,5	5078	4,22	279,6	6	1,8	-
34	08.09.27	92	1	30.04.18	1	0	0	3	3	30.04.18	1	2	2	1	3	17	0	1	2	3	2	1,8	58	17,90	-	2,38	7,02	4794	4,46	722,6	14	5,16	Fortimel compact
35	13.01.17	101	1	30.04.18	2	3	0	3	4	30.04.18	1	2	2	0	2	16	0	2	2	3	2	1,6	45	17,58	-	-	6,59	7259	4,4	-	-	-	-
36	03.04.26	92	1	30.04.18	2	0	0	3	3	01.05.18	1	4	2	0	2	11	0	4	3	5	3	1,5	42	18,67	-	2,78	6,36	5716	4,32	657,4	22	3,88	-
37	11.02.39	79	2	16.05.18	1	0	0	0	2	16.05.18	1	4	1	1	1	17	0	1	2	0	2	1,68	77	27,28	-	3,21	7,72	4352	4,64	476,6	15	8,93	-
38	24.10.29	88	2	18.05.18	1	0	0	0	3	20.05.18	1	4	1	1	3	18	0	1	2	1	3	1,7	65	22,49	-	2,03	6,37	2985	4,44	1845	9	6,29	ProvideXtra
39	03.04.47	71	1	30.05.18	1	0	0	0	2	30.05.18	1	2	1	1	1	9	0	1	2	0	2	1,65	58	21,30	-	4,14	7,56	6916	4,5	-	-	-	-
40	14.03.40	78	2	12.06.18	1	2	0	1	3	12.06.19	1	4	1	1	3	22	0	1	2	1	2	1,78	80	25,25	-	3,22	7,63	7040	4,58	446,6	19	11,86	-
41	29.08.39	78	1	15.06.18	1	3	0	2	3	15.06.18	1	2	2	1	1	17	0	1	2	2	2	1,65	70	25,71	-	-	6,86	5757	4,62	242,2	3	4,78	-
42	18.03.37	81	1	18.06.18	2	2	1	3	3	18.06.18	1	2	2	1	3	22	0	2	2	3	2	1,64	54	20,08	-	2,7	6,32	4118	4,32	300,3	34	6,11	Energea P + Fortimel Comp
43	12.02.42	76	1	26.06.18	1	0	0	0	3	27.06.18	1	4	1	1	4	15	0	1	2	0	2	1,66	74	26,85	-	2,72	7,18	9737	4,92	259,2	9	6,57	-
44	27.07.29	88	1	04.07.18	1	3	0	2	3	04.07.18	1	2	2	1	2	16	0	2	3	4	3	1,65	65	23,88	-	2,71	6,86	6166	4,66	192,7	4	2,74	-
45	21.04.26	92	2	26.06.18	1	0	0	2	2	04.07.18	1	2	2	1	3	20	0	1	2	3	3	1,74	79	26,09	-	-	6,29	5677	4,4	234,4	43	4,17	-
46	24.01.45	73	1	09.07.18	1	0	0	1	4	10.07.18	1	1	2	1	4	15	0	1	2	0	1	1,58	37	14,82	-	2,68	8,41	2830	4,64	371	21	11,02	Fortimel compact
47	02.04.41	77	1	23.07.18	1	3	1	0	3	24.07.18	1	4	2	1	3	16	0	1	2	0	2	1,74	67	22,13	-	4,23	7,06	6135	4,64	-	33	-	-
48	07.12.47	70	2	27.07.18	1	0	0	0	3	28.07.18	1	4	1	1	3	32	1	4	3	5	3	1,8	66	20,37	-	2,7	5,32	4472	4,16	240,4	9	4,73	-
49	06.11.36	81	1	01.08.18	2	2	0	3	3	01.08.18	1	5	3	1	2	19	0	4	3	5	3	1,6	70	27,34	-	2,21	6,31	3265	4,28	334,1	62	3,57	-
50	16.06.23	95	2	09.08.18	2	0	0	3	3	09.08.18	1	2	3	1	6	29	0	4	3	5	3	1,65	50	18,37		2,64	6,92	3286	4,46	279,9	9	1,99	-
51	11.01.36	82	1	12.08.18	1	0	0	2	3	13.08.18	1	4	2	1	3	17	0	1	2	2	2	1,7	82	28,37	-	3,13	7,59	11013	4,64	817,9	35	4,19	-
52	16.08.27	90	1	13.08.18	1	0	0	2	3	14.08.18	1	4	2	1	3	22	0	1	2	0	1	1,6	60	23,44	-	2,64	7,15	5760	4,76	306	9	4,5	-
53	10.02.41	77	1	15.08.18	1	0	0	3	3	15.08.18	1	4	1	1	1	20	0	4	3	5	3	1,68	45	15,94	-	2,71	6,41	5450	4,6	522,2	190	3,84	Fortimel compact
54	04.10.27	90	1	17.08.18	1	0	0	3	3	17.08.18	1	2	4	1	2	17	0	2	3	4	3	1,55	55	22,89	-	2,63	5,35	3854	4,02	161,7	32	2,28	-
55	02.05.37	81	2	19.08.18	1	2	0	2	4	19.08.18	1	2	2	1	3	15	0	1	2	3	3	1,7	78	26,99	-	2,5	6,45	7450	4,12	373,8	8	13,65	-
56	03.03.36	82	1	21.08.18	1	0	0	0	2	22.08.18	1	4	2	1	3	18	0	1	2	2	3	1,67	75	26,89	-	2,62	7,03	7580	4,7	149,5	10	4,08	-
57	30.04.27	91	1	24.08.18	2	0	0	2	2	24.08.18	1	3	3	1	2	19	1	3	3	3	3	1,5	50	22,22	-	-	6,74	6043	4,52	197,9	7	2,87	-
58	28.04.42	76	2	10.09.18	1	3	0	0	2	10.09.18	1	2	1	1	4	25	0	1	2	0	2	1,9	80	22,16	-	2,98	6,38	6268	4,5	365,2	17	8,55	-
59	04.01.29	89	2	24.09.18	1	1	0	3	3	24.09.18	1	3	2	1	6	24	0	4	3	5	3	1,83	87	25,98	6	2,76	6,94	6171	4,26	221,5	4	4,59	-
60	13.08.41	77	2	27.09.18	1	0	0	3	3	27.09.19	1	3	3	1	3	22	0	1	2	2	1	1,79	65	20,29	-	3,14	7,19	5283	4,34	262,4	7	6,82	-
61	22.07.40	78	1	02.10.18	1	0	0	3	3	02.10.18	1	5	2	1	2	33	0	2	3	3	2	1,7	75	25,95	-	3,01	7,58	6980	4,9	652,5	43	18,2	-
62	05.11.28	89	1	08.10.18	1	0	1	3	4	09.10.18	1	5	3	1	1	22	0	1	2	2	1	1,78	58	18,31	5	2,43	6,67	6045	4,62	823,3	40	27,44	Fortimel compact
63	09.06.43	75	1	12.10.18	1	0	0	0	2	12.10.18	1	1	1	1	1	19	0	1	2	0	2	1,71	58	19,84	-	3,21	7,01	7511	4,5	420,1	14	15,8	-
64	24.06.37	81	1	12.10.18	1	2	0	0	2	12.10.18	1	4	1	1	4	17	0	1	2	1	3	1,7	80	27,68	2	2,85	6,99	8455	4,4	444,6	11	10,99	-
65	02.07.47	71	1	17.10.18	1	0	0	0	3	18.10.18	1	4	2	1	4	19	0	1	2	0	2	1,64	50	18,59	-	2,9	7,86	5527	4,28	488,2	4	20	-
66	21.05.26	92	1	15.10.18	2	2	1	3	3	15.10.18	1	2	2	1	2	8	0	2	2	4	3	1,6	60	23,44	4	2,56	6,77	11350	4,66	273,5	75	3,36	-
67	11.10.18	74	1	01.11.18	2	2	0	0	3	02.11.18	1	1	2	1	5	17	0	2	2	3	3	1,6	75	29,30	2	2,48	6,78	5775	4,56	-	-	-	-
68	24.05.29	89	2	07.11.18	2	4	0	4	3	07.11.18	1	2	4	1	2	13	0	4	3	5	3	1,8	81	25,00	-	3,6	6,86	3614	4,4	265	39	1,09	-
69	18.01.41	77	2	14.11.18	1	2	0	1	3	15.11.18	1	4	2	1	1	19	0	1	2	2	3	1,82	105	31,70	-	2,79	6,87	8703	4,5	124,1	4	3,2	-
70	18.01.29	89	2	21.11.18	1	1	0	0	3	21.11.18	1	2	2	0	3	19	0	1	2	1	3	1,78	73	23,04	2	3,92	6,44	5132	4,6	378,4	38	15,92	-
71	14.08.26	92	2	22.11.18	1	2	1	2	3	22.11.18	1	2	3	1	2	23	0	1	2	3	3	1,8	73	22,53	3	3,05	7,07	8709	4,56	263,9	25	2,07	-
72	10.06.34	84	1	24.11.18	1	0	0	3	3	25.11.18	1	2	3	3	2	26	0	2	3	3	2	1,65	60	22,04	-	2,38	7,55	6407	4,78	135,8	3	6,07	-
73	16.06.35	83	1	12.12.18	1	2	0	1	3	12.12.18	1	2	2	1	1	20	0	1	2	3	3	1,52	48	20,78	2	3,04	6,74	8941	4,24	164,8	3	1,48	Fortimel compact
74	17.04.44	74	1	15.12.18	1	0	0	2	3	15.12.18	1	4	2	0	4	16	0	1	2	0	1	1,68	65	23,03	2	2,53	7,05	8734	4,48	1584	50	5,89	-
75	02.10.44	74	1	15.12.18	1	0	0	0	2	16.12.18	1	4	2	1	1	17	0	1	2	0	2	1,72	66	22,31	-	2,3	6,6	5663	4,08	400,6	7	4,52	-
76	24.05.34	84	2	21.12.18	1	0	0	3	3	21.12.18	1	2	4	1	2	20	0	2	3	4	3	1,6	60	23,44	5	2,58	6,79	5593	4,6	171,4	6	1,54	Fresubin
77	28.09.38	80	1	22.12.18	1	0	0	0	2	22.12.18	1	2	3	1	3	25	0	1	2	2	3	1,53	48,5	20,72	3	2,37	7,34	7991	4,42	199,9	9	4,84	-
78	12.07.41	77	1	01.01.19	1	2	0	0	3	01.01.19	1	2	3	1	1	20	0	1	2	2	3	1,59	84	33,23	-	2,39	7,13	10313	4,1	147,3	4	19,33	-
79	04.10.34	84	1	02.01.19	1	2	0	3	4	03.01.19	1	4	3	3	1	19	0	4	3	5	3	1,6	60	23,44	-	2,5	6,63	4922	4,38	105,7	3	3,19	-
80	09.04.36	82	2	02.01.19	1	1	0	0	3	03.01.19	1	4	1	1	4	12	0	1	2	0	2	1,76	70	22,60	-	3,04	7,21	6457	4,6	251,3	9	-	-
81	25.03.29	89	1	03.01.19	1	0	0	3	4	04.01.19	1	4	1	1	1	20	0	1	2	3	2	1,65	80	29,38	3	2,56	6,68	7028	4,66	271,4	9	2,4	Fortimel
82	26.10.31	87	1	05.01.19	1	0	1	3	4	05.01.19	1	4	2	1	3	31	0	2	3	3	2	1,58	80	32,05	3	2,55	6,78	7091	4,78	252,2	49	2,58	-
83	05.02.40	78	1	07.01.19	1	0	0	0	2	07.01.19	1	4	1	1	4	21	0	1	2	0	2	1,7	75	25,95	2	3,02	7,57	7114	4,28	320,3	13	8,11	-
84	29.01.26	92	1	13.01.19	1	2	0	1	3	13.01.19	1	2	2	1	3	24	0	1	2	2	3	1,68	62	21,97	2	-	6,57	7876	4,6	221,6	4	3,09	-
85	14.03.39	79	1	18.01.19	1	0	0	0	3	18.01.19	1	2	2	1	3	17	0	1	2	2	3	1,58	61	24,44	3	2,75	6,86	9900	4,54	389,3	3	7,54	-
86	27.03.31	87	2	18.01.19	2	4	0	3	3	18.01.19	1	2	4	0	6	11	0	4	3	5	3	1,8	70	21,60	4	3,03	6,18	5134	4,54	-	-	-	-
87	19.02.43	75	1	29.01.19	1	0	0	1	3	30.01.19	1	1	2	1	3	15	0	1	2	2	3	1,57	68	27,59	2	2,85	6,37	8046	4,54	899,5	23	17,84	-
88	14.04.29	89	2	04.02.19	1	2	0	3	3	05.02.19	1	4	2	1	3	16	0	1	2	3	2	1,6	50	19,53	2	2,7	6,97	4290	4,46	644,3	7	6,75	-
89	21.01.48	71	1	09.02.19	1	3	0	0	3	09.02.19	1	4	2	3	1	16	0	1	2	1	3	1,6	55	21,48	3	2,1	5,65	3924	4,12	300,6	12	7,24	-
90	12.01.33	86	2	13.02.19	1	0	0	0	2	14.02.19	1	3	2	3	1	21	0	1	2	1	3	1,72	75	25,35	2	2,94	6,49	6309	4,62	253,3	46	8,87	-
91	28.07.46	72	1	19.02.19	2	0	0	2	3	19.02.19	1	5	2	3	1	31	0	1	1	2	2	1,5	70	31,11	5	-	7,18	6664	4,52	164,3	3	3,99	-
92	19.08.19	99	1	21.02.19	2	0	0	2	3	21.02.19	1	2	2	1	2	26	0	2	2	3	3	1,6	55	21,48	3	3,05	6,98	7244	4,78	611,3	19	4,91	-
93	22.07.41	77	1	22.02.19	1	3	0	0	2	22.02.19	1	2	2	1	3	32	0	1	2	1	3	1,7	60	20,76	3	2,67	6,98	6425	4,62	1334	29	15,06	-
94	15.11.35	83	2	25.02.19	1	1	0	0	2	25.02.19	1	2	3	0	6	41	0	4	3	5	3	1,9	76	21,05	6	2,48	6,37	6111	4,5	2734	34	14,82	-
95	28.02.24	94	1	25.02.19	1	0	0	0	3	26.02.19	1	2	3	1	3	21	0	1	2	3	3	1,6	65	25,39	2	3,23	6,95	8086	4,54	936,5	8	6,53	-
96	10.12.39	79	1	27.02.19	1	0	1	2	3	27.02.19	1	4	2	1	3	22	0	1	2	2	2	1,55	53	22,06	3	2,63	6,56	5265	4,06	1140	49	3,65	-
97	15.02.35	84	2	01.03.19	1	0	0	4	3	01.03.19	1	2	4	0	6	6	0	4	3	5	3	1,8	80	24,69	5	3,61	7,38	6094	4,6	131	10	7,75	-
98	14.05.19	87	1	01.03.19	1	2	1	2	3	01.03.19	1	2	3	3	3	31	0	1	2	3	3	1,54	70	29,52	2	3,01	6,53	6108	4,66	752,8	24	4,95	-
99	25.07.41	77	1	04.03.19	1	1	0	0	3	04.03.19	1	2	2	0	3	24	0	1	2	0	2	1,62	82	31,25	3	2,58	7,07	9055	4,44	509,9	28	19,69	-
100	28.09.34	84	1	04.03.19	1	0	0	0	3	04.03.19	1	2	2	1	2	16	0	2	3	4	3	1,6	70	27,34	3	2,6	7,69	6506	4,44	675,3	39	30,1	-
101	27.11.20	98	1	11.03.19	1	0	0	3	2	11.03.19	1	2	3	1	1	46	0	1	2	3	2	1,55	70	29,14	4	2,81	6,54	5493	4,64	399,1	4	6,31	-
102	07.04.31	87	2	13.03.19	1	1	0	3	4	14.03.19	1	4	3	1	6	11	0	4	3	5	3	1,7	72	24,91	-	2,62	6,17	2376	4,54	-	-	-	-
103	13.09.27	91	1	14.03.19	1	2	0	3	3	15.03.19	1	3	3	1	3	30	0	1	2	3	2	1,6	58	22,66	3	2,7	7,14	6705	4,48	2552	12	6,27	-
104	03.03.30	89	2	18.03.19	1	0	0	3	3	19.03.19	1	2	3	1	1	16	0	4	3	5	3	1,68	90	31,89	2	2,64	5,96	3349	4,2	278,6	4	2,75	-
105	08.07.34	84	2	24.03.19	1	4	0	1	4	25.03.19	1	5	3	0	3	29	0	1	2	4	3	1,75	87	28,41	3	3,3	7,25	6778	4,6	457	40	6,05	-
106	07.10.31	87	2	25.03.19	1	3	1	2	3	26.03.19	1	4	2	1	3	19	0	1	2	1	1	1,62	56	21,34	-	2,81	6,68	4587	4,58	639,8	36	5,54	-
107	18.01.27	92	1	28.03.19	2	2	1	1	3	28.03.19	1	5	2	1	2	19	1	4	3	5	3	1,7	65	22,49	3	2,4	6,61	8638	4,56	866	22	4,49	Fortimel compact
108	03.05.45	73	1	29.03.19	1	0	0	0	2	29.03.19	1	2	1	1	3	19	0	1	2	2	3	1,7	68	23,53	-	2,49	6,32	5549	4,4	553,4	4	24,38	-
109	09.09.34	84	1	30.03.19	1	2	0	3	3	31.03.19	1	4	3	1	3	16	0	1	2	3	2	1,68	64	22,68	2	2,67	7,25	7572	4,72	811	15	8,5	-
110	08.08.46	72	1	31.03.19	1	0	0	0	2	01.04.19	1	4	2	1	3	15	0	1	2	2	3	1,6	69	26,95	1	3,09	7,25	10319	4,8	134,1	12	22,1	-
111	07.05.32	86	1	01.04.19	1	3	0	2	3	02.04.19	1	2	3	1	1	21	0	1	2	3	3	1,68	68	24,09	4	2,36	6,73	7928	4,28	1829	26	37,76	-
112	29.05.40	78	1	06.04.19	1	0	0	0	3	06.04.19	1	2	2	1	3	23	0	1	2	2	3	1,55	50	20,81	2	-	6,44	5536	4,34	165,6	7	7,54	-
113	15.12.31	87	1	08.04.19	1	0	0	0	3	09.04.19	1	1	2	1	3	33	0	1	2	3	3	1,7	58	20,07	5	3,19	6,28	4884	4,2	322,2	16	6,65	-
114	20.05.31	87	1	15.04.19	1	0	0	0	2	16.04.19	1	4	2	1	3	16	0	1	2	2	3	1,65	62	22,77	2	3,96	6,61	7862	4,68	479,1	12	18,82	-
115	08.08.46	72	1	02.05.19	1	3	0	2	3	02.05.19	1	2	2	3	3	18	0	1	2	3	3	1,43	40	19,56	4	2,15	7,54	6451	4,68	404,5	69	4,76	-
116	10.12.34	84	1	11.05.19	1	0	0	0	2	11.05.19	1	2	2	1	3	16	0	1	2	2	3	1,55	52	21,64	-	2,71	7,04	5238	4,36	1419	6	4,8	-
117	08.06.31	87	1	12.05.19	1	0	0	3	3	13.05.19	1	4	3	1	1	17	1	1	2	3	2	1,65	85	31,22	3	2,74	6,87	7307	5,04	352,7	4	3,77	-
118	20.08.31	87	1	19.05.19	1	3	0	3	3	19.05.19	1	3	2	1	3	23	0	1	2	3	2	1,72	74	25,01	2	2,31	6,92	7098	4,64	264,9	7	6,48	-
119	21.05.36	83	1	22.05.19	2	2	0	2	3	22.05.19	1	4	2	1	1	46	0	2	2	3	3	1,63	61	22,96	3	3,16	6,46	7004	4,26	279,5	5	5,43	-
120	03.06.40	79	1	26.05.19	1	0	0	0	3	27.05.19	1	4	2	3	3	15	0	1	2	0	2	1,63	70	26,35	3	2,77	6,52	7264	4,3	70,3	3	6,13	-
121	19.01.34	85	1	27.05.19	1	1	1	0	3	27.05.19	1	2	2	1	1	16	0	1	2	0	2	1,65	71	26,08	2	2,76	7,13	7103	4,56	745,3	24	10,46	-
122	03.12.22	96	1	29.05.19	1	0	0	4	3	29.05.19	1	5	3	1	3	20	0	1	2	3	1	1,7	70	24,22	3	2,12	7,42	10469	4,64	339,4	69	13,44	-
123	19.12.31	87	1	30.05.19	1	0	0	3	2	31.05.19	1	4	3	1	3	17	0	1	2	3	2	1,65	60	22,04	4	-	7,11	6470	4,64	123,7	3	1,48	-
124	21.10.28	90	1	01.06.19	1	2	1	2	3	01.06.19	1	4	4	1	6	2	0	4	3	5	3	1,7	45	15,57	-	2,05	6,32	6598	4,64	129,3	-	8,35	-
125	16.05.37	82	2	02.06.19	1	0	0	0	1	02.06.19	1	1	1	3	1	32	0	1	2	0	2	1,82	85	25,66	3	2,62	7,55	8080	4,44	143,7	17	8,9	-
126	07.08.39	79	1	16.06.19	1	2	0	0	3	17.06.19	1	2	3	1	3	17	0	1	2	3	3	1,7	60	20,76	3	2,77	6,58	6693	4,56	601,8	10	3,33	-

127	02.04.44	75	2	17.06.19	1	3	1	1	3	17.06.19	1	2	2	1	3	14	0	1	2	3	3	1,82	105	31,70	2	3,06	6,34	6194	4,8	296,1	27	8,96	-
128	16.03.42	77	1	16.06.19	1	2	0	3	3	17.06.19	1	4	2	1	3	15	0	1	2	2	1	1,65	56	20,57	3	2,7	6,57	5457	4,32	519,4	26	3,67	Fortimel
129	03.10.38	80	2	17.06.19	2	4	0	3	3	18.06.19	1	2	4	1	2	15	0	2	2	3	2	1,75	70	22,86	3	2,49	7,03	7232	4,66	209,7	38	2,05	-
130	02.09.29	89	1	23.06.19	1	3	0	3	3	23.06.19	1	5	2	1	3	15	0	1	2	3	2	1,6	52	20,31	3	2,75	7,02	6416	4,42	239,4	31	7,28	-
131	18.06.29	90	2	10.07.19	2	4	0	4	3	11.07.19	1	4	4	0	6	8	0	4	3	5	3	1,75	70	22,86	3	2,81	7,62	4345	4,74	-	-	-	-
132	09.06.35	84	1	11.07.19	1	2	0	2	2	11.07.19	1	2	2	1	3	19	0	1	2	2	2	1,72	68	22,99	2	3,25	6,48	4146	4,2	795,5	13	14,3	-
133	16.04.25	94	1	10.07.19	1	0	0	2	3	11.07.19	1	4	2	1	3	17	0	1	2	3	3	1,64	58	21,56	3	2,64	7,72	8879	4,5	86,2	8	6,48	Fortimel
134	18.12.35	83	2	13.07.19	1	2	1	0	3	13.07.19	1	2	1	1	1	16	0	1	2	0	2	1,78	78	24,62	2	3,34	6,76	5840	4,32	206,5	27	5,58	-
135	14.08.24	94	1	25.07.19	1	1	0	1	3	26.07.19	1	4	2	1	3	16	0	1	2	2	3	1,64	50	18,59	4	2,83	7,29	3710	4,46	1514	75	6,78	-
136	30.11.27	91	1	27.07.19	1	0	0	2	3	27.07.19	1	2	3	1	6	22	0	4	3	5	3	1,6	58	22,66	3	2,36	7,12	5976	4,48	310,8	4	3,12	-
137	17.11.42	76	2	29.07.19	1	2	0	0	3	30.07.19	1	2	3	3	3	22	0	1	2	2	3	1,92	81	21,97	3	2,44	7,45	8366	4,64	175,6	10	2,51	-
138	17.05.30	89	1	02.08.19	1	2	1	3	3	03.08.19	1	4	2	1	3	17	0	1	2	2	1	1,7	76	26,30	-	2,93	7,34	5916	4,5	1619	26	27,36	-
139	13.02.49	70	2	16.08.19	1	0	0	0	2	16.08.19	1	2	2	1	1	10	0	1	2	3	3	1,9	60	16,62	4	2,41	7,35	5553	4,8	191,6	28	4,18	Fresubin
140	21.05.34	85	1	16.08.19	1	2	0	3	3	16.08.19	1	3	3	3	1	17	0	1	2	3	2	1,75	60	19,59	4	3,7	6,6	7875	4,82	145,5	20	3,07	Diben
141	25.10.43	75	1	16.08.19	2	2	1	2	3	17.08.19	1	2	2	1	2	9	0	2	2	3	3	1,63	65	24,46	4	3,52	6,42	4521	4,86	337,4	13	4,36	-
142	26.07.30	89	1	20.08.19	1	1	1	3	4	20.08.19	1	2	2	1	2	15	0	1	2	4	3	1,55	55	22,89	2	2,29	7,42	5300	4,58	1008	63	13,56	-
143	11.08.43	76	1	23.08.19	1	2	0	0	3	23.08.19	1	4	2	1	1	23	1	1	2	2	3	1,65	55	20,20	3	4,53	7,96	7675	5,26	1036	49	24,15	-
144	26.06.35	84	1	02.09.19	2	2	0	1	3	02.09.19	1	3	2	0	3	16	0	1	1	2	3	1,6	60	23,44	3	2,38	7,85	8135	4,72	1168	38	16,9	-
145	09.11.37	81	1	02.09.19	1	2	0	0	3	03.09.19	1	4	1	1	3	15	0	1	2	1	3	1,75	72	23,51	3	2,82	6,79	6151	4,78	475,9	24	3,92	-
146	31.10.28	90	1	03.09.19	2	0	0	0	2	03.09.19	1	3	2	1	3	14	0	1	1	3	3	1,58	63	25,24	3	2,85	6,48	7075	4,44	419,9	18	5,13	-
147	24.10.29	89	2	14.09.19	1	0	1	0	3	15.09.19	1	4	2	1	3	19	0	1	2	0	2	1,65	70	25,71	2	2,24	6,32	6140	4,46	582,1	37	16,8	-
148	08.12.40	78	1	16.09.19	1	1	1	2	3	16.09.19	1	2	2	1	1	18	0	1	2	3	3	1,55	55	22,89	3	2,78	7,02	5612	4,6	293,9	27	4,86	-
149	21.06.23	96	1	18.09.19	1	0	1	2	3	18.09.19	1	2	2	1	3	16	0	1	2	2	2	1,6	70	27,34	-	2,67	7	10257	4,8	244,4	45	4,51	-
150	09.04.25	94	1	20.09.19	1	0	0	0	2	20.09.19	1	2	2	1	3	61	0	1	2	2	3	1,6	60	23,44	3	2,69	6,17	8097	4,54	398,2	6	3,94	Fortimel
151	10.06.47	72	1	22.09.19	1	1	3	0	3	23.09.19	1	4	4	3	3	26	0	1	2	0	2	1,59	92	36,39	3	2,06	6,88	9306	4,78	1028	30	6,42	-
152	16.03.31	88	1	30.09.19	2	3	0	3	3	01.10.19	1	3	4	0	2	15	0	4	3	5	3	1,65	70	25,71	3	1,84	7,26	6279	4,56	911	3	5,21	-
153	08.03.22	97	1	14.10.19	1	2	1	3	3	15.10.19	1	4	4	0	2	23	0	4	3	5	3	1,7	65	22,49	4	2,39	6,09	5708	4,78	336,3	60	7,95	Cubitan
154	08.08.45	74	1	17.10.19	1	2	3	2	2	17.10.19	1	3	1	3	1	13	0	1	2	2	2	1,52	73	31,60	-	-	6,68	5733	4,42	-	-	-	
155	26.07.29	90	1	19.10.19	1	3	1	3	3	19.10.19	1	3	3	1	3	23	0	2	3	3	2	1,65	65	23,88	3	2,05	6,8	6284	4,6	927,7	56	3,81	
156	13.07.30	89	1	21.10.19	2	0	1	3	3	21.10.19	1	2	2	1	2	15	0	4	3	5	3	1,54	50	21,08	2	2,81	6,16	5257	4,16	144,7	83	2,77	
157	23.10.44	75	1	25.10.19	2	3	0	3	3	25.10.19	1	2	4	1	2	18	0	2	2	4	3	1,65	80	29,38	-	2,17	7,07	11212	4,78	262,4	38	5,67	
158	30.11.25	93	1	31.10.19	1	0	0	2	3	01.11.19	1	2	2	3	3	17	0	3	3	2	2	1,6	55	21,48	3	2,65	6,37	7460	4,64	237,7	3	8,38	
159	26.08.29	90	2	02.11.19	1	0	0	0	3	03.11.19	1	2	2	3	1	18	0	1	2	2	3	1,71	70	23,94	3	2,84	6,1	4128	4,5	278,1	10	3,69	
161	22.01.26	93	1	05.11.19	1	3	0	2	3	06.11.19	1	4	2	3	3	15	0	1	2	2	2	1,65	60	22,04	-	2,65	7,83	7073	5,1	-	-	-	
162	08.09.28	91	1	11.11.19	1	3	0	3	3	11.11.19	1	2	2	3	3	22	0	2	3	2	1	1,59	49	19,38	-	2,68	8,27	7122	5,1	307,8	23	4,93	
163	05.01.34	85	1	14.11.19	1	3	1	0	3	14.11.19	1	2	2	3	1	20	0	1	2	2	3	1,65	73	26,81	3	2,42	7,08	5403	4,88	291,9	27	3,39	
164	15.12.46	72	2	16.11.19	1	0	1	3	3	16.11.19	1	2	3	3	3	24	0	1	2	2	1	1,85	66	19,28	-	-	7,61	4683	4,84	440,3	39	30,62	
165	10.12.28	90	1	17.11.19	1	0	1	2	2	17.11.19	1	3	2	3	3	20	0	2	3	2	2	1,6	50	19,53		2,49	7,1	6014	4,58	257,3	36	4,07	
166	21.01.31	88	1	16.11.19	2	3	0	3	3	17.11.19	1	2	4	0	6	3	0	4	3	5	3	1,63	49	18,44	6	3,11	6,73	4988	4,76	-	-	-	
167	24.06.23	96	1	22.11.19	2	2	0	3	3	22.11.19	1	2	3	1	3	35	0	4	3	5	3	1,6	45	17,58	2	2,51	7,08	5575	4,32	1143	3	2,21	
168	13.12.24	94	2	24.11.19	1	1	1	3	4	25.11.19	1	4	4	0	6	2	0	4	3	5	3	1,8	80	24,69	-	2,09	6,11	3348	4,18	-	-	-	
169	10.05.31	88	1	25.11.19	1	3	0	3	3	26.11.19	1	4	2	1	1	16	0	1	2	2	1	1,62	60	22,86	-	2,77	6,75	7246	4,28	319,2	7	9,85	-
170	06.07.37	82	1	28.11.19	2	3	0	3	3	29.11.19	1	4	2	3	3	17	0	1	1	2	1	1,68	85	30,12		2,85	6,29	9515	4,42	682,1	13	7,53	-
171	28.05.33	86	2	18.12.19	2	3	1	3	3	18.12.19	1	2	3	1	2	16	0	2	2	4	3	1,85	90	26,30	3	-	6,33	9523	4,48	-	-	-	-
172	24.01.38	81	2	02.01.20	1	2	0	0	3	03.01.20	1	4	1	3	3	17	0	1	2	2	3	1,74	80	26,42	2	2,63	6,46	4890	4,26	808,1	4	7,44	-
173	01.01.37	83	1	13.01.20	1	3	0	2	3	15.01.20	1	5	3	1	3	46	0	1	2	2	2	1,6	79	30,86	2	3,11	7,13	5533	4,42	576,1	16	3,38	-
174	01.06.23	96	1	15.01.20	2	2	1	3	3	15.01.20	1	2	3	1	2	16	0	2	2	3	2	1,65	60	22,04	-	2,66	6,54	6743	4,64	175,2	27	3,72	-
175	07.10.37	82	1	15.01.20	1	0	0	0	2	16.01.20	1	2	1	3	3	15	0	1	2	0	2	1,63	60	22,58	2	2,92	6,53	6857	4,3	169,5	3	7,02	-
176	18.06.35	84	1	15.01.20	1	4	0	2	3	16.01.20	1	4	2	1	2	17	0	1	2	2	2	1,68	60	21,26	2	-	6,35	3559	5,16	1282	31	4,17	-
177	18.07.37	82	1	19.01.20	1	1	0	0	2	20.01.20	1	3	2	1	3	43	0	1	2	0	2	1,72	70	23,66	2	2,67	4,94	3545	3,9	190,5	14	2,8	-
178	24.09.37	82	1	20.01.20	1	2	0	2	3	20.01.20	1	2	2	3	1	24	0	1	2	2	2	1,54	75	31,62	2	3,06	7,08	10438	4,48	329,2	20	10,32	-
179	19.11.38	81	1	20.01.20	1	0	0	2	2	20.01.20	1	2	3	0	1	18	0	1	2	3	3	1,72	68	22,99	3	2,72	7,21	7341	4,68	338,3	3	4,98	Fortimel
180	22.05.24	95	2	22.01.20	1	2	1	3	4	22.02.20	1	3	2	1	1	16	0	4	3	5	3	1,82	90	27,17	2	2,57	6,16	2724	4,44	1225	54	4,5	-
181	21.10.36	83	2	24.01.20	1	3	0	2	3	24.01.20	1	4	4	0	6	30	0	4	3	5	3	1,75	80	26,12	5	2,64	4,57	3072	3,86	423,5	7	3,79	-
182	29.11.39	80	2	30.01.20	1	3	1	2	2	30.01.20	1	4	3	1	1	19	0	1	2	3	3	1,82	67	20,23	3	2,5	7,76	6081	4,64	397,3	23	8,43	-
183	22.07.40	79	1	30.01.20	1	0	0	0	2	31.01.20	1	4	4	3	1	17	0	1	2	0	2	1,7	79	27,34	2	-	8,17	7533	4,68	464	16	9,06	-
184	18.09.72	77	2	31.01.20	1	4	0	0	3	31.01.20	1	4	3	3	3	36	0	1	2	3	3	1,82	85	25,66	2	3,54	5,88	4975	4,08	535,8	5	4,35	-
185	16.03.35	84	2	31.01.20	2	0	0	0	3	31.01.20	1	4	3	1	2	15	1	4	3	5	3	1,765	75	24,08	3	3,17	6,19	6246	4,34	277,5	11	8,81	-
186	06.08.22	97	1	08.02.20	2	2	0	2	3	08.02.20	1	5	3	1	2	20	0	4	3	5	3	1,47	45	20,82	3	-	6,42	3735	4,32	243,9	34	3,89	-
187	24.01.34	86	2	13.02.20	2	3	1	3	3	14.02.20	1	4	3	1	1	14	1	1	1	4	3	1,82	83	25,06	3	2,69	6,06	4179	4,06	767,7	49	6,08	-
188	25.07.25	94	1	14.02.20	1	2	1	2	3	15.02.20	1	4	4	0	6	3	0	4	3	5	3	1,65	64	23,51	5	2,73	7,2	8720	4,78	1383	25	10,69	-
189	22.03.23	96	1	24.02.20	1	0	0	2	3	24.02.20	1	4	2	1	3	16	0	1	2	3	3	1,65	72	26,45	2	4,29	7,48	9301	4,8	329,2	41	5,13	-
190	24.06.29	90	1	24.02.20	1	3	0	2	3	24.02.20	1	2	3	1	6	27	0	4	3	5	3	1,76	78	25,18	3	2,59	7,05	5549	4,4	240,2	13	2,57	-
191	23.02.32	88	2	25.02.20	1	3	1	2	3	25.02.20	1	2	2	1	3	14	0	1	2	2	2	1,73	73	24,39	2	2,99	6,04	4815	4,36	293,9	16	3,41	-
192	25.06.40	79	1	27.02.20	1	0	0	0	1	27.02.20	1	2	2	3	3	20	0	1	2	3	3	1,77	95	30,32	2	-	7,77	7759	4,64	411	15	12,67	-
193	02.06.39	80	1	01.03.20	1	0	0	0	3	02.03.20	1	4	2	1	3	15	0	1	2	3	3	1,64	65	24,17	2	-	6,98	6389	4,48	373	9	8,59	-
194	18.11.39	80	1	04.03.20	1	3	1	2	3	04.03.20	1	2	4	1	2	21	0	4	3	5	3	1,67	57	20,44	3	3,77	7,2	8235	4,66	-	-	-	Fortimel
195	17.10.38	81	1	04.03.20	1	4	0	3	4	05.03.20	1	4	4	1	1	22	0	1	2	3	2	1,65	90	33,06	2	3,74	5,99	4644	3,94	291,3	3	4,12	-
196	30.05.33	86	1	06.03.20	2	2	1	4	3	06.03.20	1	2	4	1	2	10	0	2	2	4	2	1,65	55	20,20	4	2,82	6,94	3975	4,28	194,5	39	2,29	Fortimel
197	31.01.36	84	1	11.03.20	1	0	0	4	3	11.03.20	1	2	4	1	1	15	0	1	2	4	2	1,7	50	17,30	5	2,36	6,99	5516	4,46	166,4	4	2	Fortimel+Cubitan
198	12.01.20	100	1	25.03.20	1	0	0	2	2	25.03.20	1	2	2	1	3	22	0	2	3	4	3	1,63	45	16,94	3	3,13	7,1	5911	4,78	90,3	3	8,65	Fortimel
199	12.03.29	91	1	24.03.20	2	3	1	2	4	25.03.20	1	4	4	0	6	8	0	4	3	5	3	1,6	60	23,44	3	3,02	6,2	5715	4,38	560,8	55	90.3	-
200	16.11.39	80	1	26.03.20	1	1	0	3	3	26.03.20	1	2	3	1	1	14	0	4	3	5	3	1,6	45	17,58	4	2,22	7,31	8910	4,86	1713	3	5,21	Fresubin
201	01.11.34	85	1	30.03.20	1	0	0	2	3	31.03.20	1	4	2	1	3	15	0	1	2	2	2	1,72	60	20,28	3	2,24	5,98	6297	4,38	866,9	19	17,31	-
202	12.02.39	81	2	04.04.20	1	2	0	3	3	05.04.20	1	2	4	1	5	25	0	2	3	3	2	1,75	65	21,22	4	2,36	7,56	3562	4,74	322,9	11	7,47	Fortimel
203	27.10.36	83	2	05.04.20	1	0	0	2	3	06.04.20	1	4	2	0	3	20	0	1	2	2	2	1,65	80	29,38	7	2,28	7	6323	4,46	379,7	13	4,42	-
204	15.08.36	83	2	09.04.20	1	3	1	1	3	09.04.20	1	2	1	1	6	22	0	4	3	5	3	1,8	84	25,93	2	2,15	6,81	6680	3,94	162,1	25	7,11	-
205	19.05.34	85	1	12.04.20	1	0	0	2	3	13.04.20	1	2	2	1	3	16	0	4	3	5	3	1,65	60	22,04	3	3	7,7	7533	4,62	315,3	3	7,81	Fortimel
206	24.01.45	75	2	14.04.20	2	3	0	2	3	14.04.20	1	3	2	1	3	19	0	2	2	2	2	1,7	78	26,99	2	2,92	7,2	3505	4,3	357,5	17	6,42	-
207	23.08.32	87	2	18.04.20	1	1	0	1	3	19.04.20	1	4	2	0	3	22	0	1	2	2	3	1,7	90	31,14	3	2,65	7,2	6193	4,46	482	37	7,56	-
208	24.05.39	80	1	19.04.20	2	0	0	3	3	20.04.20	1	2	3	1	3	16	0	2	2	2	1	1,58	51	20,43	5	2,19	6,89	5797	4,54	167,4	47	2,62	Fortimel
209	27.01.38	82	2	26.04.20	1	0	0	3	3	26.04.20	1	2	3	1	3	38	0	2	3	4	3	1,73	75	25,06	2	2,89	7	8695	4,38	168,8	12	5,36	-
210	25.10.48	71	1	28.04.20	1	2	0	0	2	29.04.20	1	2	1	3	4	16	0	2	3	0	2	1,55	76	31,63	2	-	6,31	8125	4,64	206,4	8	5,06	-
211	13.03.39	81	1	02.05.20	1	4	0	1	3	02.05.20	1	2	1	0	1	16	0	1	2	1	2	1,7	63	21,80	2	3,96	7,53	6863	4,66	-	34	-	-
212	06.04.36	84	1	02.05.20	1	0	0	0	3	03.05.20	1	4	3	3	1	21	0	1	2	2	3	1,65	70	25,71	3	3,16	7,48	6778	4,64	364,8	7	4,79	-
213	02.12.26	93	1	06.05.20	1	2	1	0	3	06.05.20	1	2	2	1	3	19	0	2	3	1	3	1,68	80	28,34	2	3,28	6,91	5986	4,44	193,3	37	4,52	-
214	22.08.34	85	1	07.05.20	1	0	0	1	3	08.05.20	1	4	2	3	3	18	0	2	3	1	2	1,7	69	23,88	2	2,94	5,62	4712	4,3	244,3	38	10,19	-
215	18.04.29	91	1	09.05.20	2	0	1	2	3	10.05.20	1	4	2	1	2	15	1	2	2	2	2	1,65	68	24,98	3	3,03	8,51	7025	4,82	214,8	22	7,72	-
216	27.12.38	81	1	12.05.20	2	0	0	3	3	13.05.20	1	4	4	1	2	15	0	4	3	5	3	1,7	72	24,91	-	2,25	5,74	6397	4,36	161,2	11	2,8	-
217	01.12.38	81	1	12.05.20	1	0	1	2	3	13.05.20	1	4	2	1	3	22	0	4	3	5	3	1,62	62	23,62	2	2,36	7,56	6776	4,98	234,6	23	9,51	Fortimel
218	25.09.24	95	1	15.05.20	2	0	1	3	3	16.05.20	1	4	2	3	2	18	0	2	2	2	1	1,65	65	23,88	2	2,07	6,58	5791	3,72	184,4	30	8,4	-
219	11.02.42	78	1	16.05.20	1	2	0	0	3	16.05.20	1	3	2	3	3	16	0	1	2	0	2	1,73	52	17,37	3	2,01	6,4	4493	4,44	321,3	9	15	-
220	15.06.39	80	1	21.05.20	1	0	0	0	3	22.05.20	1	4	1	1	3	17	0	1	2	0	2	1,68	57	20,20	3	-	6,93	6109	4,66	367,1	58	3,77	-
221	20.09.36	83	1	22.05.20	1	3	0	3	3	23.05.20	1	2	2	3	3	22	0	2	3	0	1	1,65	65	23,88	3	2,47	8,05	5649	4,94	204,9	8	3,24	-
222	10.09.49	70	1	02.06.20	1	0	0	0	3	02.06.20	1	2	1	1	3	15	0	1	2	0	2	1,58	63	25,24	-	-	7,38	8030	4,76	110,3	16	8,95	-
223	18.02.25	95	1	06.06.20	1	0	0	0	3	06.06.20	1	2	4	1	6	3	0	4	3	5	3	1,6	50	19,53	3	2,87	7,12	4347	4,44	76,9	31	13,03	-
224	10.09.33	86	2	10.06.20	1	0	0	0	3	10.06.20	1	2	2	1	3	15	0	1	2	1	3	1,83	90	26,87	2	2,58	6,77	6506	5,18	249,8	10	10,11	-
225	22.04.39	81	1	16.06.20	1	3	0	1	4	16.06.20	1	2	2	1	1	19	0	1	2	2	3	1,65	66	24,24	2	2,2	6,4	6959	4,82	-	-	-	-
226	11.01.47	73	1	19.06.20	1	0	0	0	3	20.06.20	1	1	2	0	1	6	1	1	2	2	3	1,68	63	22,32	-	3	6,1	6917	4,98	1832	14	2,69	-
227	02.02.37	83	1	27.06.20	1	0	0	0	2	28.06.20	1	3	2	1	3	21	0	1	2	1	3	1,75	95	31,02	3	2,33	6,92	4753	4,64	177,6	20	9	-
228	16.07.39	80	1	30.06.20	1	0	0	0	2	30.06.20	1	2	1	3	3	16	0	1	2	2	3	1,63	73	27,48	2	3,08	6,36	7559	4,36	235,7	5	10,35	-
229	01.12.30	89	2	04.07.20	1	0	0	1	2	04.07.20	1	2	2	3	3	19	0	1	2	4	3	1,8	65	20,06	2	2,08	7,18	5621	4,64	327	10	-	Fortimel/Fresubin
230	03.09.27	92	1	13.07.20	2	3	1	0	3	13.07.20	1	2	4	1	6	7	0	4	3	5	3	1,64	45	16,73	-	2,66	7,31	2533	3,78	568,5	3	2,99	-
231	24.06.35	85	2	16.07.20	1	0	1	3	3	16.07.20	1	2	3	1	1	19	0	1	2	4	3	1,67	90	32,27	2	1,9	6,78	3915	4,38	347,7	10	8,61	Fortimel
232	13.07.29	91	2	19.07.20	1	0	0	1	3	19.07.20	1	2	3	1	1	17	0	1	2	2	3	1,81	74	22,59	-	2,43	7,07	8113	4,32	142,5	7	11,31	-
233	28.12.37	82	1	19.07.20	1	2	0	0	3	20.07.20	1	1	1	3	1	14	0	1	2	1	3	1,63	61	22,96	-	2,59	7,85	8690	4,62	307,7	6	17,53	-
234	21.08.37	82	1	25.07.20	1	3	0	0	3	25.07.20	1	2	2	1	3	17	0	1	2	2	3	1,6	59	23,05	2	3,16	7,61	6487	4,6	218,6	41	6,25	-
235	30.01.30	90	2	29.07.20	1	3	0	0	3	30.07.20	1	1	1	1	3	16	0	1	2	2	3	1,71	74	25,31	1	-	6,51	4933	4,6	256	5	4,03	-
236	25.07.30	90	1	03.08.20	2	2	0	1	3	03.08.20	1	2	3	1	2	14	0	2	2	2	3	1,46	49	22,99	-	2,57	6,33	7313	4,34	161,5	29	3,01	-
237	05.11.39	80	1	07.08.20	2	2	0	2	3	07.08.20	1	2	3	1	2	20	0	2	2	3	3	1,54	50	21,08	3	2,19	6,71	5274	4,74	519,2	4	3,93	Fortimel
238	18.09.31	88	2	14.08.20	1	4	0	3	3	14.08.20	1	2	3	1	1	11	0	1	2	4	3	1,8	70	21,60	2	3,01	5,88	5506	4,24	262,2	16	6,44	-
239	03.01.49	71	2	21.08.20	1	2	0	0	3	21.08.20	1	2	2	1	1	18	0	1	2	1	3	1,76	83	26,79	2	-	7,38	5536	4,78	211,2	13	8,91	-
240	05.08.24	96	1	24.08.20	1	2	1	1	3	25.08.20	1	1	3	3	2	14	0	1	2	2	3	1,76	50	16,14	5	3,64	6,63	6040	4,6	1746	15	30	Fortimel
241	22.11.34	85	1	24.08.20	1	0	0	0	3	25.08.20	1	1	2	3	1	14	0	2	3	1	3	1,58	70	28,04	-	2,72	6,44	6427	4,46	253,7	23	6,46	-
242	02.12.30	89	2	26.08.20	1	0	1	0	3	26.08.20	1	2	2	1	1	7	0	1	2	2	3	1,7	65	22,49	3	2,65	5,57	2744	4,1	243,8	17	-	-
243	27.03.41	79	1	29.08.20	1	3	0	0	3	30.08.20	1	2	2	3	3	23	0	1	2	2	3	1,65	62	22,77	2	2,83	6,93	6188	4,68	147,7	13	2,79	-
244	21.02.37	83	1	04.09.20	2	0	3	4	2	05.05.20	1	2	4	1	2	16	0	2	2	4	2	1,58	55	22,03	2	2,76	5,49	5235	5,12	197,2	44	2,72	-
245	29.04.41	79	1	14.09.20	1	1	2	0	3	14.09.20	1	3	2	2	4	15	0	1	2	1	3	1,65	64	23,51	2	2,43	7,63	8129	4,2	461,9	32	9,11	-
246	28.09.40	79	2	13.09.20	1	3	0	0	3	14.09.20	1	4	1	1	4	15	0	1	2	0	2	1,83	92	27,47	2	2,67	6,59	5338	4,44	553,6	8	9,76	-
247	05.07.46	74	1	16.09.20	1	0	0	0	2	17.09.20	1	4	1	3	1	14	0	2	3	2	3	1,7	58	20,07	5	-	7,14	7731	4,92	390,2	25	13,26	-
248	05.12.35	84	2	17.09.20	2	3	0	0	4	17.09.20	1	2	3	1	2	15	0	4	3	5	3	1,82	58	17,51	7	2,34	6,24	3038	4,6	-	-	-	Fortimel
249	17.05.25	95	1	18.09.20	2	0	0	1	3	18.09.20	1	2	3	3	3	17	0	2	2	3	3	1,5	55	24,44	5	2,46	7,4	12928	4,38	185,7	5	3,16	-
250	04.01.43	77	2	20.09.20	2	0	0	3	4	21.09.20	1	4	3	0	2	23	0	2	2	1	1	1,75	75	24,49	3	2,87	7,05	6304	4,56	1009	14	18,03	Fortimel
251	24.08.33	87	1	25.09.20	1	0	0	0	3	25.09.20	1	2	2	1	3	18	0	1	2	1	3	1,62	56	21,34	3	3,18	7,79	6855	4,78	791	46	11,45	-
252	15.02.35	85	1	30.09.20	1	3	0	2	3	30.09.20	1	2	3	3	1	34	0	1	2	2	2	1,67	70	25,10	2	2,84	5,87	3687	5,06	487,2	36	7,79	Fortimel
253	10.02.35	85	2	01.10.20	1	0	0	0	3	01.10.20	1	2	2	3	1	18	0	1	2	0	2	1,8	86	26,54	2	2,77	6,25	5140	4,24	349,1	15	13,74	-
